# Policy mix for a low-carbon food system

**DOI:** 10.3389/fnut.2026.1766609

**Published:** 2026-03-31

**Authors:** Marta Buso, Sanchayan Banerjee, Thales A. P. West

**Affiliations:** 1 Institute for Environmental Studies, Vrije Universiteit Amsterdam, Amsterdam, Netherlands; 2 School for Government, The Policy Institute, King's College London, London, United Kingdom

**Keywords:** carbon offset, carbon pricing, ETS, nudge, sequencing, nudge+

## Abstract

The current food system accounts for approximately a third of the global greenhouse gas emissions, necessitating an urgent shift toward low-carbon food production and consumption practices. Carbon pricing can help internalize the negative externalities of deriving animal-based protein. There have been suggestions to complement meat and dairy taxes with softer policy tools, such as behavioral nudges, that instead encourage people to adopt plant-based diets. Nonetheless, both hard and soft tools have limitations when applied in isolation—taxation is perceived to be unpopular, while softer tools might be ineffective. In this perspective, we argue that innovations to this policy toolkit are key for a food systems transformation. Our proposition includes novel hard policy tools, such as the Emission Trading System (ETS), and seemingly softer policy tools, such as Voluntary Carbon Offsetting (VCO), both of which can be combined with softer tools like nudge and nudge+ interventions. We argue that policy innovations, as well as sequencing soft and hard tools, could enhance existing food policies, by creating a more socially acceptable pathway toward the urgently needed transition to a sustainable food system, thereby improving the effectiveness of these policies.

## Introduction

The current food system accounts for one-third of global greenhouse gas emissions (GHG) (1) and poses significant challenges for biodiversity, ecosystem services, and air pollution (2) as well as human health (3). Animal-based food accounts for 32% of CO_2_ emissions, while plant-based food contributes to 19% of carbon emissions (4). Additionally, global population growth and increased per capita meat consumption are expected to boost livestock production, further exacerbating these externalities (5).

The price of livestock products does not reflect the harm they cause (6). This is in contrast to the “polluter-pay-principle”, which suggests pricing goods to reflect social damages at the margin (7). Internalizing the negative externalities of animal-based products should be addressed at the global scale, due to the transversality of the negative effects of climate change. In this perspective we propose policies mix and sequencing that can be contextualized and adapted for each region's specificities.

Recently, meat taxes have been suggested as one of the solutions to internalize the damaging effects of meat production and consumption (8–10). Denmark has been the only country to implement a tax on agricultural emissions (11). Habib et al. (12) explore the positive and negative implications of applying carbon pricing along the food supply chain. They argue that carbon pricing policies pose various challenges, such as higher costs and administrative burden, GHG emissions leakage and regressive impacts on consumers.

Carbon pricing policies can be categorized as “hard” policy instruments, which involve the restriction of personal choices and the use of mandatory financial interventions; in contrast, “soft” policies provide information to the public and guide their decisions through behavioral interventions (13, 14). This is also known as the Nuffield Intervention Ladder for public health policies (13). Soft policies are popular tools to steer citizens toward more sustainable and healthy diets, which can considerably reduce the environmental impact of our food system (15). For example, nudges have been applied to encourage sustainable food choices (16) and reduce meat consumption (17). Hess et al. (18) found nudges to be more effective than a competency-building boost in a food-delivery environment, while Banerjee et al. (19) argue that encouraging reflection on dietary choices reduced food emissions of food orders by 40%. Nevertheless, soft interventions might sometimes backfire and lead to an increase in meat consumption (17, 20). Given the negative impacts that both types of policy may generate, more comprehensive policy measures and a more accepted sequence of policy mix are needed.

Are there any other tools that we can employ to reduce livestock emissions? In this perspective, we move along the hard and soft carbon pricing policy Nuffield ladder (13) to discuss other potential fiscal tools, specifically cap-and-trade Emissions Trading Systems (ETS) and Voluntary Carbon Offsets (VCOs). We consider how hard policies can be combined with behavioral interventions such as “nudge+”—which incorporates a reflective component into a standard nudge—to overcome some of their limitations when applied in the food system, and how hard, soft and combined policies should be sequenced to be more acceptable.

## Emissions Trading Systems (ETS)

ETS are commonly based on the cap-and-trade principle, where operators can trade allowances to emit CO_2_-eq, corresponding to emissions reductions in relation to a cap defined by regulators for specific sectors. For example, the European Union (EU) ETS, the largest ETS in operation, covers GHG emissions from the energy, manufacturing industry and aircraft sectors, but not agriculture (21). Discussions of expanding the ETS also to the agriculture sector have started in the European Commission (22). The True Animal Protein Price (TAPP) Coalition (23) proposed to include slaughterhouses and dairy factories in a new Agri-ETS system. However, industry lobbying might hinder its implementation, as it has already influenced the EU GHG market design in the past (24).

A report published by the European Commission (25) analyzed the implications of implementing the EU ETS at different points of the food system value chain: livestock, arable and mixed farms; manufacturers and importers of farm animal feed and synthetic fertilizer; processors of meat and dairy products. Among the five options proposed in the report, there is a downstream GHG emissions tax that lies with entities that receive meat and dairy products sold by farmers. An ETS applied to only enterprises with more than 50 people would cover almost 82% and 91% of the total turnover of processing and preserving meat and dairy products in the European Union (25). However, mandatory fiscal policies will continue to face strong opposition from lobbyists from the ultra-processed food industry (26) and farmers (27), which could further lower actions to tackle livestock emissions.

Along with the pushback from lobbyists, the inclusion of agricultural sectors in the EU ETS would likely come with drawbacks that policymakers should consider. Among these, there is the expected administrative burden on small farmers — 9 out of 10 of EU farms are family farms (28). While some parties proposed to exempt small farmers from the ETS system, others defend the promotion of agricultural cooperatives to share the administrative burden and costs or foster the digitalisation of the monitoring and reporting processes (25, 29). Another potential issue is GHG emissions leakage (30). Farms and firms might find it more lucrative to relocate their businesses where carbon regulations are less stringent or absent. Therefore, we argue that a food-related ETS system should not be implemented alone, but in harmony with a set of European-wide policies that avoid potential backlash on the economy. The Carbon Border Adjustment Mechanism (CBAM) is a tool that aims at preventing these negative effects (31). The CBAM imposes a price on carbon-intensive imported goods from countries with less stringent environmental regulations, preventing carbon leakage from the European Union (31).

## Voluntary Carbon Offsetting (VCO)

Given the limitations and barriers of implementing an ETS in the agriculture sector, the real cost of livestock products could be slowly introduced with voluntary fiscal tools. A recent EU legislation launched a voluntary Union certification framework for permanent carbon removals and carbon farming (CRCF; 31). Operators can be eligible for a voluntary certification for any activities implemented that reduce their emissions level in the EU (32). An additional voluntary alternative could be represented by voluntary carbon offsetting, offering polluters the opportunity to compensate for their emissions by subsidizing GHG emission reductions achieved by mitigation projects elsewhere through the purchase of carbon credits in voluntary or compliance carbon markets (33). This could be applied along the whole value chain — even at the consumer level — to compensate for the negative environmental impacts of their consumption choices. VCO have been extensively studied in the aviation sector, and the willingness to pay for VCO has been applied to delivery services (34), conference (35), electricity consumption (36), and touristic activities (37). The UN implemented a platform where individuals can compute their carbon footprint and contribute to UN programmes that reduce GHG emissions (38). At the firm level, in the UK, the impact of food products has been claimed to be offset via sustainability projects (39).

Nevertheless, while VCO could be the backbone of more attractive and convenient soft policy alternatives, there are large uncertainties about their effectiveness (40–43), posing a considerable obstacle for implementation and societal acceptance (44). In that regard, emphasis could be placed on VCO activities based on more credible assumptions and calculations, such as those involving the destruction of methane emissions in landfills of SF_6_ and HFC-23 (45). Still, even if more reliable options are available, this should not distract society from a permanent shift toward more sustainable food choices.

Another issue related to VCO refers to moral licensing (46). Individuals and firms might alleviate guilt feelings while continuing to engage in unsustainable consumption practices. If VCO is to be promoted as part of a soft policy tool within the food system, regulators must ensure, to a reasonable degree, that they correspond to actual emissions reductions (47) and do not delay global decarbonization efforts. Whether this target is realistically achievable has yet to be determined (48).

## Policy innovations

We visualize the interventions mentioned above on the Nuffield Intervention Ladder: carbon pricing policies, as the meat-tax and the Agri-EU ETS, are placed among the hard policies, on top of the ladder, behavioral tools are positioned among the soft policies, at the bottom of it and carbon offsetting would be placed in the middle. We suggest that rather than moving from one type to the other, implementing a combination of soft and hard policies^1^ might make carbon pricing more effective. For example, Wang and Li (49) argue that soft economic incentives and behavioral incentives are more effective than individual policy alone. An example of a union of these policies is Compensators e.V., (50) a platform which buys allowances to withdraw them from the EU ETS market. This is a combination of a hard instrument (i.e., cap-and-trade market) and a soft tool. Other authors explored the possibility of blending behavioral instruments with carbon pricing policies. Gravert and Shreedhar (51) proposed combining carbon tax policies and green nudges to create a more salient choice environment that evokes climate concern, targets multiple behaviors and increases public acceptance. In contrast to traditional nudge, which changes the choice architecture without changing the economic incentives or the choice set (52), green nudges aim to promote sustainable behaviors (53, 54). Framing is also an example of this combination of soft and hard policy tools. For example, consumers are particularly sensitive to how taxation is framed; Baranzini and Carattini (55) argue that adopting the term “climate contribution,” rather than “tax,” could increase public support.

The effectiveness of voluntary soft tools can be enhanced by a reflection prompt. In this regard, VCO could be implemented along with nudge+, a behavioral tool that incorporates a reflection trigger inside the traditional nudge (56). For example, in the context of food purchase, customers could be asked whether they would like to pay an additional amount to offset their food emissions, with the opportunity to scan a QR code or a visual prompt that explains how carbon offsetting works and what climate change mitigation activity they will support. While consumers can make more sustainable food choices with more effective information-based interventions (57), we are aware that this is context-dependent. When individuals decide what to consume, they do it in a choice environment which is already filled with information (e.g., vegetarian or vegan labels, allergens, and nutritional attributes). In addition, according to the context in which the decision is taken, different thinking processes might be operating. Kahneman (58) defined two specific thinking processes: System 1, fast, automatic and intuitive; and System 2, slower and complex reasoning. In some contexts, such as food delivery platforms or restaurants, where people tend to have more time to make decisions, a VCO with a nudge+ might be easier to implement. It is important to adjust the type of behavioral tool according to which system is involved in the decision-making process. In settings such as supermarkets or cafeterias, a reflective tool would be rarely feasible; therefore, a behavioral tool that focuses on the faster, automatic and intuitive System 1 will likely be more effective. Studies that combine VCO with behavioral tools, e.g., Zheng et al. (59), include the use of heuristics to induce people to purchase carbon offsets, although the former authors found that transparent information was more effective than heuristic cues. Another option is using anchors, i.e., when people make their decision by adjusting their choice to an initial piece of information (60), to influence people's offsetting decision (61), or setting offsetting as a default decision option (62).

## Discussion and conclusions

Stepping from softer to harder policies might be a suitable approach to help individuals become accustomed to pro-environmental behaviors, before forcing them to adopt them with more coercive (i.e., harder) policies. However, the public's attitudes on policies differ according to the level of coerciveness, the level of power the state is exerting, the administrative design distinction and the salience of the policy (63). A policy mix might increase acceptability across individuals' political orientation and policy preferences (63). Meckling et al. (64) described how California and the EU adopted a sequence of policies for a low-carbon energy transition, moving from green industrial regulation to a carbon-pricing initiative to a policy mix (64). We propose a similar approach, by sequencing from soft policies to a combination of soft and hard policies. For example, VCO, as a voluntary fiscal measure, might introduce the idea of accounting for the real price of animal-based products when environmental (and health) externalities are considered. It could be introduced as a first step in a dietary fiscal policy agenda at the downstream level of the agricultural sector (processors of meat and dairy products), with potential marketing benefits for environmentally conscious customers. This might steer firms in the food production industry to implement more sustainable solutions in their business model (e.g., production shift to avoid offsetting) and avoid a long-term reliance on VCO. At the same time, nudge+ could be more extensively implemented at the consumer level as soft initial measures (e.g., engaging in consumers' sustainable choices using websites and apps) or included in harder policies to steer reflection on the potential loss associated with harmful behavior (e.g., financial loss derived from food waste). This would tackle their environmental attitudes and beliefs to start, which is the first step to induce a more long-lasting behavior and a habit (65).

We argue for the use of different soft tools simultaneously throughout the food supply chain to create a more comprehensive policy mix that can gain public support. These measures would be voluntary at first, focused on changing views and beliefs, while pushing firms to a new, more sustainable food system. As a following step, more stringent policies should come into force, e.g., an Agri-ETS or a mandatory carbon pricing in the form of a carbon tax, framed as a “levy” (66) or “climate contribution” (55), along with nudging measures, reinforcing the reasons and needs for more stringent climate measures. Soft tools as nudge or nudge+, could be useful instruments for addressing hurdles associated with “harder” climate policies, such as economic costs, distributional dynamics and lack of capacity and authority and free-riding concerns (67). Revenues from carbon taxes could also be used to subsidize healthier and plant-based products, to avoid a regressive effect of the tax (68–70). The price reduction of these products could be made more salient using behavioral measures.

While some of these combinations of soft and hard tools (such as framing) entail less administrative burden, they are not free of potential bureaucratic issues arising from policy mixes. In the EU context, some mechanisms have already been deployed to reduce the bureaucratic burden (71). We believe that a fair and acceptable policy mix should be designed to not hinder the law-making process and overburden citizens with more red tape.

Synergies between these hard and soft measures require further research, and they might be a potential tool to move toward more sustainable dietary choices. Nevertheless, these tools need to be tailored according to the contexts in which they are applied. An active participation from all the stakeholders involved, including citizens, might help policymakers to design fairer and more effective policy interventions. Public involvement is pivotal to introducing acceptable and stringent climate policies (72). We believe that recent behavioral innovations, such as nudge+, can ease citizen engagement in public policy initiatives by allowing citizens to become autonomous and enter a mature dialogue with the state. Citizen engagement could also be achieved through citizens' assemblies, which can encourage public participation in the early stages of policymaking (73). Building such participatory public policies will help adapt and refine the policy mix as presented in this perspective according to the citizens' needs and the specific socioeconomic context.

## Data Availability

The original contributions presented in the study are included in the article/supplementary material, further inquiries can be directed to the corresponding author.

## Footnotes

1 Public policies vary in their levels of stringency. Depending on the level of stringency they can be classified either as soft (such as education, persuasion, information provision) or hard (command-and-control or fiscal regulations such as taxes); for details, see Banerjee et al., (14).
